# Emergence and Evolution of High-Level Cephalosporin-Resistant *Salmonella* Goldcoast in Northern Taiwan

**DOI:** 10.1093/ofid/ofz447

**Published:** 2019-12-17

**Authors:** Ye Feng, Yi-Jung Chang, Szu-Hsuan Fang, Lin-Hui Su, Hsin-Chieh Li, Hsin-Ping Yang, Min-Jia Yu, Cheng-Hsun Chiu

**Affiliations:** 1 Sir Run Run Shaw Hospital, Zhejiang University School of Medicine, Hangzhou, China; 2 Institute for Translational Medicine, Zhejiang University School of Medicine, Hangzhou, China; 3 Key Laboratory of Microbial Technology and Bioinformatics of Zhejiang Province, Hangzhou, China; 4 Molecular Infectious Disease Research Center, Chang Gung Memorial Hospital, Chang Gung University College of Medicine, Taoyuan, Taiwan; 5 Division of Pediatric Infectious Diseases, Department of Pediatrics, Chang Gung Memorial Hospital, Chang Gung University College of Medicine, Taoyuan, Taiwan

**Keywords:** cephalosporin resistance, clone, conjugative plasmid, *Salmonella* Goldcoast, whole-genome sequencing

## Abstract

**Background:**

Nontyphoidal *Salmonella* (NTS) is an important foodborne pathogen worldwide. We investigated a 2018 outbreak of highly antimicrobial-resistant *Salmonella enterica* serotype Goldcoast in northern Taiwan.

**Methods:**

We collected 30 clinical isolates and 2 meat isolates from this outbreak in New Taipei and Taoyuan, Taiwan in 2018. The clinical manifestations and the treatment of the patients were reviewed. To trace the source, we examined NTS isolated from food samples collected from the markets in northern Taiwan. All of the isolates along with an additional human isolate from China were sequenced and compared with the sequences of *Salmonella* Goldcoast reported by other countries.

**Results:**

The outbreak involved 14 pediatric patients (<5 years old) and 16 adults (36 to 83 years old). Nine patients with invasive or severe disease required carbapenem treatment. The MIC_90_ of ceftriaxone and ciprofloxacin for the outbreak isolates was >256 μg/mL and 1 μg/mL, respectively, and a conjugative 278-kilobase plasmid harboring *bla*_CTX-M-55_ and *qnrS1* contributed towards the resistance. Whole-genome sequencing revealed a clonal relationship among the outbreak isolates and the 2 collected from the retail meats. The outbreak clone was phylogenetically close to that of *Salmonella* Goldcoast reported in the United Kingdom, Poland, and China, whereas similar resistance plasmids were found in China and Cambodia.

**Conclusions:**

The clinical spectrum of the high-level cephalosporin-resistant *Salmonella* Goldcoast is similar to that of other NTS serotypes, but severe cases required carbapenem treatment. The study confirmed the emergence of a highly antimicrobial-resistant clone of *Salmonella* Goldcoast, highlighting the importance of surveillance for food safety.

## Teaser

An outbreak caused by ceftriaxone- and ciprofloxacin-resistant *Salmonella* Goldcoast was investigated. A conjugative 278-kb plasmid harboring *bla*_CTX-M-55_ and *qnrS1* contributed to the resistance. Whole-genome sequencing revealed a clonal relationship among the clinical isolates and another 2 from retail meats.

Nontyphoidal *Salmonella* (NTS) is a prevalent foodborne pathogen that usually causes gastroenteritis, but it can occasionally cause invasive diseases in humans [1, 2]. It has been estimated that there are more than 90 million cases of gastroenteritis due to NTS globally each year, with 85% of those cases being linked to foods consumed [3]. More than 2500 NTS serotypes have been identified, but only a few dozen, particularly *Salmonella* Typhimurium and *Salmonella enteritidis*, accounted for 50%–70% of NTS infections worldwide [4–6].

Salmonellosis caused by NTS is usually self-limiting, but when antimicrobial treatment is necessary, ampicillin and trimethoprim-sulfamethoxazole were the drugs of choice in the clinical setting. Antimicrobial-resistant NTS has emerged since the 1980s, in which the ACSSuT-resistant phenotype (defined as resistant to ampicillin, chloramphenicol, streptomycin, sulfamethoxazole, and tetracycline) was first identified in *Salmonella* Typhimurium DT104 in England [7, 8]. This penta-resistance pattern is mediated by a Class I integron, which is usually associated with the mosaic structure of the *Salmonella* genomic island 1 (SGI1) [9]. Due to the rapid dissemination of ACSSuT resistance in *Salmonella*, fluoroquinolones (FQs) and extended-spectrum cephalosporins are recommended for treating NTS infections in such settings. In 2017, the susceptible rate of *Salmonella* to ceftriaxone and ciprofloxacin in Taiwan was 92.3% and 78.7%, respectively [10].

*Salmonella* Goldcoast, a rare serotype for human infection, belongs to serogroup C2. To date, all reported foodborne outbreaks linked to *Salmonella* Goldcoast have occurred in Europe, with the contamination source being French pate, watercress, cheddar cheese, and raw fermented sausages [11–14]. The *Salmonella* Goldcoast strains recovered from these outbreaks were mostly sensitive to FQs and cephalosporins. In 2018, we experienced an *Salmonella* Goldcoast outbreak in Taiwan. This emerging *Salmonella* Goldcoast clone exhibited high-level resistance to extended-spectrum cephalosporins and concurrent resistance to FQs. Moreover, although the genomic analysis demonstrated that the outbreak isolates showed a close clonal relationship with strains isolated from the United Kingdom and China, the conjugative resistance plasmid was highly similar to the plasmids circulating in China and Cambodia. In this study, we report the clinical and microbiological investigation of this outbreak in northern Taiwan and highlight the importance of monitoring antimicrobial resistance (AMR) of foodborne pathogens.

## METHODS

### Patients and Setting

**Figure 1.**
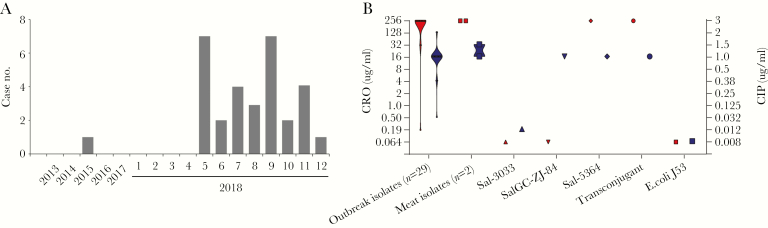
*Salmonella* Goldcoast infection in Taiwan and antimicrobial resistance of the isolates. (A) Number of the *Salmonella* Goldcoast cases in Chang Gung Memorial Hospital (CGMH). Between January 2013 and April 2018, only 1 case was reported, whereas between January 2018 and December 2018, 30 cases were identified. (B) Violin plot of the minimum inhibitory concentration (MIC) values of ceftriaxone ([CRO] in red, left y-axis) and ciprofloxacin ([CIP] in blue, right y-axis) for the studied isolates. Isolate Sal-3033 was the only one collected at CGMH before 2018. Isolate SalGC-ZJ-84 was collected from Zhejiang, China instead of Taiwan. The MICs for the donor strain, Sal-5364, the recipient strain, *Escherichia coli* J53, and the transconjugant obtained in the conjugation experiment demonstrate that the resistance was mediated by a conjugative plasmid.

Chang Gung Memorial Hospital (CGMH) is a main referral hospital for cities in northern Taiwan, including Taipei, New Taipei, and Taoyuan. The population in this region is approximately 7 million. The Clinical Microbiology Laboratory has launched a program to monitor the serotypes of the NTS causing human infections since 2012. All *Salmonella* isolates derived from patients were collected and serotyped. Antimicrobial susceptibility testing was performed using the disc diffusion method specified in the Clinical and Laboratory Standards Institute (CLSI) guidelines. *Salmonella* Goldcoast is a relatively uncommonly recorded serotype for human infections. Before 2015, only 1 *Salmonella* Goldcoast isolate was recovered from the stool of a child with acute gastroenteritis. From 2016 to 2017, no *Salmonella* Goldcoast was further reported. However, in 2018, 30 culture-confirmed infections were found to have been caused by *Salmonella* Goldcoast (Figure 1A; Supplementary Table 1). This outbreak is still ongoing in 2019: there were already 20 cases of *Salmonella* Goldcoast infections by the end of July in 2019. Therefore, an outbreak investigation was conducted, including an analysis of the following: clinical manifestations, treatments, and patient outcomes; bacterial source tracing; and microbiological and genomic examinations of all isolates of *Salmonella* Goldcoast. Only 1 sample per patient was collected for downstream analyses. If both blood and stool isolates were available from the same patient, only the blood isolate was analyzed. Pulsed-field gel electrophoresis (PFGE) was performed by the Centers for Disease Control and Prevention in Taiwan, following the protocol as described [15]. The minimum inhibitory concentration (MIC) of ceftriaxone and ciprofloxacin to these isolates were determined by E-test and interpreted according to the recommendations given by CLSI [16]. The Student’s *t* test, the χ ^2^ test, and Fisher’s exact test or analysis of variance were used for comparisons whenever appropriate. All statistical analyses were 2-sided, and significance was set at *P* < .05.

To trace the source of *Salmonella* Goldcoast, we obtained *Salmonella* isolates from food samples collected from markets in Linko District, New Taipei City and Kweishan District, Taoyuan City. These 2 districts, with their high population densities, are 2 main areas of origin for the patients treated in CGMH. As with the clinical isolates, the *Salmonella* isolates obtained from food samples were further examined for their serotypes. Antimicrobial susceptibility to ceftriaxone and ciprofloxacin for these isolates were assessed using E-test strips.

### Genome Sequencing and Assembly

In addition to the 31 *Salmonella* Goldcoast isolates collected from CGMH (30 isolates collected in the 2018 outbreak and 1 in 2015), 1 *Salmonella* Goldcoast isolate from the neighboring Zhejiang province, China (SalGC-ZJ-84), which was collected from the stool of an 80-year-old patient with acute gastroenteritis in June 2017, was also included in this study. All of these isolates were subjected to whole-genome sequencing (WGS).

Genomic deoxyribonucleic acid (DNA) of all the sequenced isolates was prepared using the QIAamp DNA Mini Kit (Qiagen, Düsseldorf, Germany) and then subjected to WGS using the Illumina Miseq platform (Illumina, San Diego, USA). The short reads generated were de novo assembled into contigs using SPAdes version 3.11.1 with the “--careful” option [17].

The isolate, Sal-5364, was subjected to sequencing using both Illumina Miseq sequencer and the long-read MinION Sequencer (Nanopore, Oxford, UK). A de novo hybrid assembly of the short Illumina and long MinION reads was performed using Unicycler version 0.4.79 under the “normal” mode [18]. The complete circular contigs generated were then corrected using Pilon version 2.14 10 with Illumina reads [19].

### Genomic Analysis

The multilocus sequence typing (MLST) and prediction of the AMR genes were performed by analyzing the WGSs through BacWGSTdb [20]. To search for genetically close isolates from the Taiwan outbreak isolates, the global *Salmonella* Goldcoast isolates were downloaded from Enterobase (https://enterobase.warwick.ac.uk/) and NCBI GenBank database (Supplementary Table 2). An initially quick phylogeny construction was carried out using parsnp software [21].

The core genome MLST (cgMLST) was performed also using the BacWGSTdb service [20]. In brief, the cgMLST alleles were determined first, and then the allele matrix was put into GrapeTree software for construction of minimal spanning tree [22]. For single-nucleotide polymorphism (SNP) calling, the complete genome sequence of Sal-5364 was used as a reference; the query genomes were mapped against the reference genome by the nucmer program in MUMmer package version 3.22 [23]; the show-snps program was then used to call the SNPs, using the “-C” option to remove the SNPs from the alignments with ambiguous mapping. The 5-base pair adjacent SNPs were further filtered; then, Gubbins software was used to filter recombinant SNPs. An ML tree built using RAxML version 8.0.0 [24] was assessed with TempEst version 1.5.1 [25]. Given the strong temporal signal found in the data, we proceeded with time-calibrated Bayesian phylogenetic inference using BEAST version 1.10.4 [26]: the “HKY” substitution model, the “strict molecular clock” model, the “Coalescent: Constant size” tree prior model, and 10 000 000 Markov chain Monte Carlo (MCMC) chains were set for this analysis.

Similar plasmids to pSal-5364 were searched using the BacWGSTdb single-genome analysis module. In brief, BacWGSTdb collects all of the complete plasmid genomes and applies the MASH to compare them with the user uploaded sequences [27].

### Conjugation Experiment

To investigate the transferability of the resistance plasmid identified in the genome sequencing, a conjugation assay was conducted using Escherichia *coli* J53 (a sodium azide-resistant strain) as the recipient. An outbreak *Salmonella* Goldcoast isolate (Sal-5364) was conjugated with the J53 at the donor-to-recipient ratio of 1:4 and coincubated for 12 hours on a 0.45-μm microporous membrane. Transconjugants were subsequently selected on Mueller-Hinton agar supplemented with sodium azide (200 μg/mL) and ceftriaxone (16 μg/mL). S1 nuclease-PFGE was performed to determine the plasmid profile. Genomic DNA preparations for J53, Sal-5364, and transconjugant strains were made in agarose plugs and digested with S1 nuclease, and then separated by PFGE. The conjugation efficiency was measured and calculated following the protocol in https://openwetware.org/wiki/conjugation.

### Data Availability

The genome sequences sequenced in this study have been deposited in the NCBI GenBank database, and their accession numbers are listed in Supplementary Table 1.

## RESULTS

### Outbreak Description

Since May 2018, a sharp rise in infections caused by *Salmonella* Goldcoast has been observed; as of December 2018, 30 cases have been recorded (Figure 1A). All of the 14 pediatric patients were younger than 5 years of age, whereas the age of the adult patients ranged from 35 to 85 years (Supplementary Table 3). The most common symptoms noted in these patients were diarrhea, abdominal cramps, and fever (Supplementary Table 4). Most patients had only gastroenteritis, whereas 3 adults and 1 child were complicated with bacteremia, 1 adult with urinary tract infection, and 1 child with ileus. The extra-intestinal infection rate for *Salmonella* Goldcoast was 7.1% (1 of 14) for children less than 5 years of age and 25.0% (4 of 16) for adults. Pediatric patients showed a significantly longer hospital stay for treatment than did adults (*P* = .002) (Supplementary Table 4).

All isolates from this outbreak were 100% resistant to ampicillin, chloramphenicol, and trimethoprim-sulfamethoxazole. More importantly, all but 1 isolate were simultaneously resistant to ciprofloxacin and ceftriaxone. The MIC of ciprofloxacin and ceftriaxone for these isolates were mostly 1 μg/mL and >256 μg/mL (Figure 1B). Nine patients, including the 5 with extra-intestinal infections and the 1 with ileus, received carbapenem treatment, mainly with ertapenem.

### Resistance Mechanism

**Figure 2.**
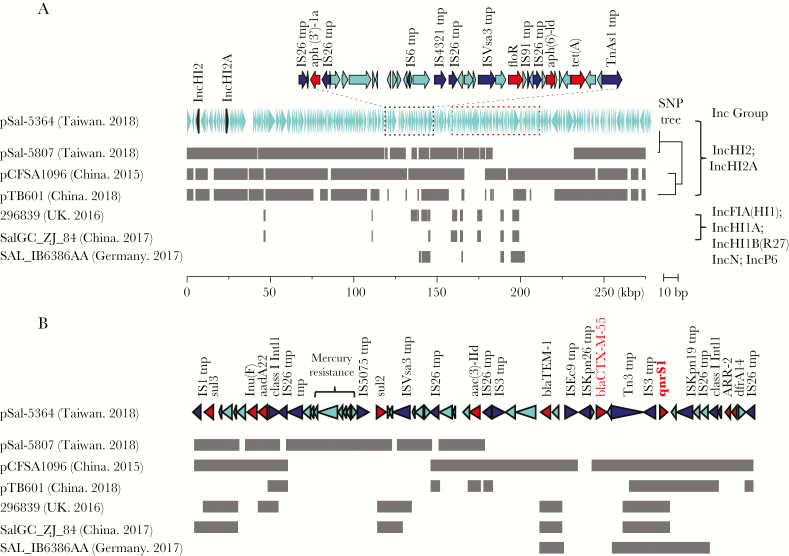
Alignment between the 278-kilobase plasmid and its closely related plasmids. The aligned regions are represented as gray blocks. (A) Sal-5364 and Sal-5807 were isolated from the outbreak; the former was resistant to ciprofloxacin and ceftriaxone, whereas the latter was susceptible to both. The replicon genes are marked in black. The resistance genes are concentrated within 2 cassettes (marked in dashed black and red boxes). The plasmids, pCFSA1096 (accession no. CP033347.1) and pTB601 (accession no. CP034832.1), revealed the same Inc group to the pSal-5364, but they did not originate from the *Salmonella* Goldcoast. To the right is the neighbor-joining tree built based on the single-nucleotide polymorphisms (SNPs) identified within the backbones of these plasmids. Isolate 296839 (accession no. SRR7204491) and isolate SAL_IB6386AA (accession no. ERR2984265) were the only European and American isolates that carried *qnrS1* in the public database. Isolate SalGC_ZJ_84 from Zhejiang, China also carried *qnrS1*. These 3 plasmids belonged to different Inc groups from the others. (B) Gene structure of the red cassette (A).

The *Salmonella* Goldcoast isolates were all subject to WGS. The resistant isolates invariably carried a 278-kilobase (kb) plasmid that belonged to the IncHI2/IncHI2A group. All resistance genes were clustered with 2 cassettes on the plasmid (Figure 2A). Insertion sequences (IS) and transposons were enriched in the 2 cassettes, indicative of their high frequency of lateral transfer. One of the 2 cassettes was bracketed by 2 integrases of the Class I integron and carried *bla*_CTX-M-55_ and *qnrS1* simultaneously. This Class I integron-like element harbored *sul2*, *sul3*, *lnu*(F), *aadA22*, *ARR-2*, and *dfrA14*, and therefore conferred the ACSSuT phenotype to the bacteria. The only susceptible isolate from this outbreak, Sal-5807, also possessed this plasmid but lacked *bla*_CTX-M-55_ and *qnrS1* (Figure 2B). An earlier isolate, Sal-5303, collected in 2015, was susceptible to ceftriaxone and ciprofloxacin due to its lack of the plasmid. The SGI1 was absent in all the *Salmonella* Goldcoast isolates.

To investigate the transferability of the resistance plasmid, conjugation experiments were carried out and revealed that the 278-kb plasmid was readily transferred into the recipient (Supplementary Figure 1), with a conjugation efficiency being 1.76 ± 1.02 × 10^‒4^. The transconjugant revealed the same level of resistance to ceftriaxone and ciprofloxacin as the donor strain and other outbreak isolates (Figure 1B), suggesting that the resistance was laterally transferrable between bacteria through plasmid conjugation.

### Phylogeny Construction and Source Identification

**Figure 3.**
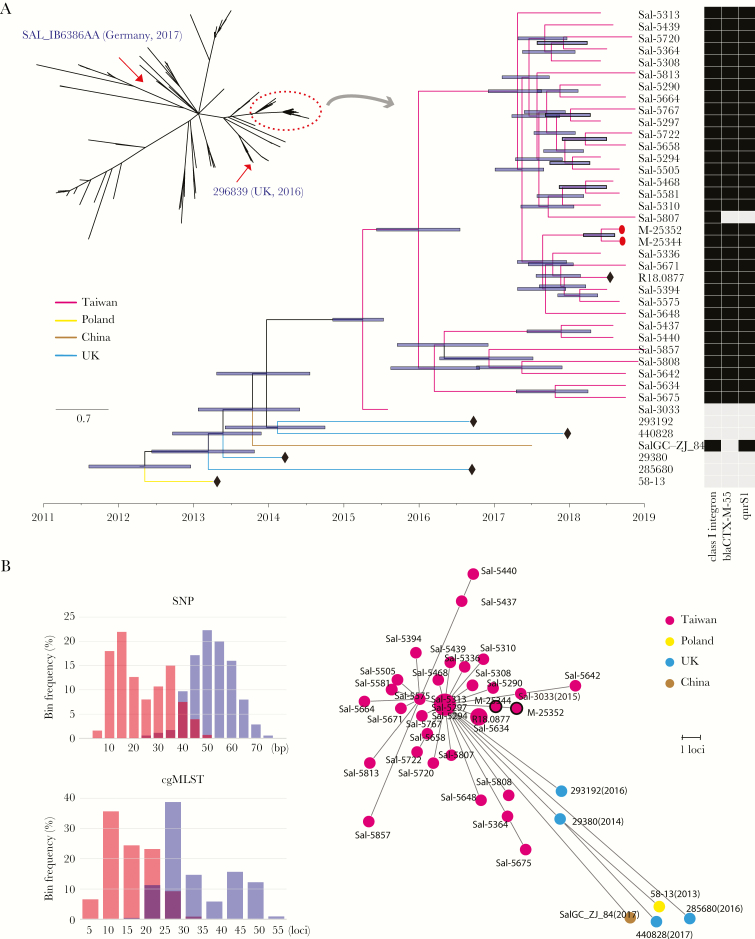
Phylogenetic analysis of the *Salmonella* Goldcoast outbreak isolates in Taiwan. (A) The phylogenetic relationship among global *Salmonella* Goldcoast isolates. The dendrogram built by parsnp software revealed that all of the Taiwanese isolates were clustered together with a few international isolates (marked in a red dashed circle). All isolates outside the red circle were in lack of *bla*_CTX-M-55_ and *qnrS1*, except the 2 isolates marked by the red arrows (see the detailed gene structure in Figure 2). A dated phylogeny was further built for the isolates within the red circle by the BEAST2 software. The branches are in different colors according to their geographic location. The black nodes in the diamond represent the data downloaded from public database, whereas the rest were sequenced in this study. The nodes in the form of red dots represent isolates of the food origin, whereas the rest were derived from the patients. The right heatmap represents the presence (in black) or absence (in gray) of key antimicrobial resistance genes. (B) Histograms of single-nucleotide polymorphism (SNP) distance and core genome multilocus sequence typing (cgMLST) distance. The pairwise distance within the Taiwanese outbreak isolates is marked in red, whereas that between the Taiwanese outbreak isolates and the international ones is in blue. (C) Minimal Spanning Tree built by GrapeTree software based on cgMLST alleles. The number in the parenthesis represents the collection year; isolates without this were all collected in 2018. Dots with black circles represent food isolates, whereas the others are clinical isolates.

All isolates belonged to sequence type 358 (ST358) and had the same PFGE pattern. Whole-genome sequencing further demonstrated that they possessed a high level of similarity, with their chromosomal difference ranging from 7 to 46 SNPs (Figure 3). This result indicated that this outbreak had originated from the same contaminated foods. To trace the source of *Salmonella* Goldcoast, we obtained food samples from traditional markets in New Taipei and Taoyuan and identified 2 *Salmonella* Goldcoast isolates from retail meats, 1 being from pork and the other from chicken (Supplementary Table 5). Whole-genome sequencing showed that the isolates from the retail meats were closely connected with the clinical isolates (Figure 3A). The retail meat isolates harbored the same 278-kb plasmid and showed the same resistance profile. Taken together, the contaminated retail meats were considered a possible source of the outbreak.

One susceptible isolate, Sal-5303, which was collected earlier in 2015, was highly homologous to the outbreak isolates in genome sequence, with its distance from the other genomes ranging from 20 to 37 SNPs. A dated phylogeny dendrogram revealed that the isolate was at the root of the outbreak (Figure 3A).

### Worldwide Dissemination of the *Salmonella* Goldcoast Clone

To detect the epidemiological and evolutionary signals from the global context, we compared the genomes of the Taiwan isolates with the 150 *Salmonella* Goldcoast genomes deposited in the GenBank, NCBI, including 44 from North America, 105 from Europe, and 1 from Taiwan. Because of the large geographic distances between the above isolates and the Taiwanese outbreak clone, we also collected and sequenced an *Salmonella* Goldcoast isolate from Zhejiang province, China, which was found 600 km across the strait from Taiwan.

As a result, a Taiwanese isolate in the public database, which had also been collected in 2018, was found nearly identical to the isolates studied (Figure 3B), suggesting that the outbreak caused by the *Salmonella* Goldcoast clone was not limited to northern Taiwan. It is interesting to note that the Zhejiang isolate sequenced, along with 4 British and 1 Polish isolates (collected in 2013–2016), were phylogenetically closely related to the Taiwanese outbreak clone, with a difference of 23–74 SNPs (Figure 3B). This distance range overlapped with that of the Taiwanese clone. The cgMLST presented similar results (Figure 3B and C). Thus, the Taiwanese isolates could not be distinguished from these international isolates purely by genetic distance, but they formed a topologically cohesive cluster. This close genetic distance demonstrated a short divergence history: the Taiwan isolates had not diverged from the international isolates until 2014 (Figure 3A).

The international *Salmonella* Goldcoast isolates were overall much more susceptible when compared with the Taiwan outbreak isolates. All of the international isolates lacked *bla*_CTX-M-55_ or other β-lactamase-encoding genes that can confer resistance to extended-spectrum cephalosporins. Almost all of them likewise lacked *qnrS1* as well as effective mutations in their chromosomal quinolone resistance-determining regions, suggesting that they would be quinolone susceptible. Only the Zhejiang isolate as well as a British and a German isolate carried *qnrS1*. As with the Taiwanese clone, these isolates’ *qnrS1* genes were carried by an IS*3* transposase but belonged to different plasmid types, suggesting that the resistance genes were carried on an entirely different plasmid (Figure 2). The p.T57S mutation in *parC* was found in all *Salmonella* Goldcoast isolates, even those susceptible to FQs. No other point mutation was found. Thus, this mutation was merely a genetic marker for this serotype but did not contribute towards resistance. For other antimicrobial agents, the Class I integron was detected in the Zhejiang isolate and 12 European isolates from among the international isolates, indicating their potential to express the ACSSuT phenotype.

The conjugative nature of the 278-kb plasmid demonstrated its transmission route as being independent from that of the chromosome. We consequently developed an online service integrated in the BacWGSTdb database [20], which applies the MASH algorithm to compare the query sequence to all of the complete plasmid sequences deposited in the NCBI database. The closest plasmids to the 278-kb resistance plasmid of *Salmonella* Goldcoast were also from the *Salmonella* species isolated in China (Figure 2); the backbones of the plasmids were similar, and the differences were mainly distributed in mobile elements. In particular, the plasmid pCFSA1096 (accession no. CP033347.1), collected from the Hubei Province, China in 2015, also carried *bla*_CTX-M-55_ and *qnrS1*.

## DISCUSSION

Raw food products are popular in Taiwan. In combination with the location’s optimum temperature range for bacterial growth, NTS infections have long been rampant on this island. The present study reported an outbreak of highly antimicrobial-resistant *Salmonella* Goldcoast and confirmed that the offending *Salmonella* was from local retail meats. The entire meat production and sale chain consists of the following 4 stages: breeding and farming, slaughter, distribution, and sale. Although a much larger body of literature has focused on isolation rates on farms and in slaughter houses, contamination may occur more commonly at the distribution and sale phase [28]. In this study, we attributed the present outbreak to the contamination occurring at the distribution and sale phase rather than during farming and slaughter. We also speculated, based on the following epidemiological and genomic evidence, that this contamination had taken place for years. First, the sampled chicken and pork came from different farms but were sold at the same market. Second, the early *Salmonella* Goldcoast isolate collected in 2015 in the same geographic region belonged to the same clone as the outbreak isolates. Third, the genomes of the outbreak isolates were not exactly identical but differed from each other by a limited number of SNPs, indicating the presence of a regional source rather than point source pollution. One limitation of this study is that we did not conduct tests upon local farms, although the farms raising pigs and chicken were likely the source of the outbreak clone. This hypothesis could not be totally rejected. We propose that actions such as transportation by cold chain, fresh meat preservation, and the improvement of sanitation in traditional markets should be undertaken to reduce the contamination and thereby avoid the occurrence of subsequent foodborne infection.

Phylogenetically, genome comparison has revealed an unexpectedly limited genetic distance between the *Salmonella* Goldcoast isolates of Taiwan and other countries in Asia and Europe. The SNP distance within the Taiwan isolates overlapped with that between Taiwan and other international isolates, making it difficult to distinguish them merely by SNP distance. This situation also applies to the cgMLST strategy. Furthermore, the genetic distance between the Taiwanese and Chinese *Salmonella* Goldcoast isolates was not shorter than that between Taiwan and British isolates; this disproportion between the SNP and spatial distances strongly suggests the involvement of human activity, such as increases in global travel and food trade, upon the transmission of the infectious disease. The ancestor for all Taiwanese *Salmonella* Goldcoast isolates can be traced to as late as 2014. However, few cases, even sporadic ones, caused by this rare serotype, have been reported in Taiwan before 2014, whereas all previously reported cases of *Salmonella* Goldcoast have been concentrated in Europe. The global dissemination of a real bacterial clone, rather than a certain PFGE type or ST, has been reported for a number of other bacterial pathogens [29]. Therefore, increased awareness, timely global communication, and collaboration for food safety are essential towards containing foodborne disease outbreaks.

Salmonellosis is usually a self-limiting disease that does not require antimicrobial therapy, but in young children and the elderly population, if the offending *Salmonella* causes an invasive illness, antimicrobial therapy becomes necessary. Because the ACSSuT penta-resistance has reached 14.5%, 72.7%, and 28.8% in the United States, Taiwan, and China [30], respectively, FQs and extended-spectrum cephalosporins became the antimicrobials of choice for the treatment of salmonellosis. Before 2003, resistance to FQ was mainly attributed to an accumulation of mutations in such bacterial genes as *gyrA* and *parC* targeted by the drug. For example, the *Salmonella* Choleraesuis outbreak isolates in Taiwan in 2000 were highly resistant to FQ (>8 μg/mL) by their simultaneous carriage of F83S and N87D mutations in *gyrA* [31]. A single T57S mutation in an entire *Salmonella* Goldcoast lineage is not sufficient for a strain to achieve clinically significant resistance, but 1 additional mutation could result in highly FQ resistance. Meanwhile, during the past 15 years, there have been increasing reports of FQ-resistant Enterobacteriaceae encoding *qnrA*, *qnrB*, *qnrD*, aac(6’)-Ib-cr, and *oqxAB*, as well as *qnrS1* [32], the gene responsible for the FQ resistance in *Salmonella* Goldcoast. These plasmid-mediated quinolone resistance genes generally facilitate low-level FQ resistance, but their potential to be laterally transferred promotes their rapid dissemination.

Growing resistance of NTS to extended-spectrum cephalosporins is usually linked to the spread of plasmid-mediated AmpC or extended-spectrum β-lactamase (ESBL) genes. The most common global contributor is *bla*_CMY-2_, followed by *bla*_CTX-M-15_ [33–36]. As a variant of *bla*_CTX-M-15_, *bla*_CTX-M-55_ increases the catalytic efficiency of the ESBL to cephalosporins, leading to a higher degree of resistance than *bla*_CTX-M-15_. The *bla*_CTX- M-55_ gene has already become prevalent in China, Thailand, and Cambodia [30, 37–42], but the present study is the first case reported in Taiwan. The 2 resistance genes, namely, *bla*_CTX-M-55_ and *qnrS1*, were both carried by a 278-kb conjugative plasmid in *Salmonella* Goldcoast outbreak strains. In this regard, surveillance and monitoring of the plasmid’s circulation among the Enterobacteriaceae species is crucial. Nowadays, a few databases, such as Enterobase (https://enterobase.warwick.ac.uk) and BacWGSTdb (http://bacdb.org/BacWGSTdb), have been constructed with the aim of investigating genetic relatedness based on genomic sequences and, accordingly, establishing epidemiological links between bacterial pathogens. Few databases, on the other hand, have been developed for plasmids. To help address this concern, we developed an online service for plasmid tracing. We have discovered highly similar plasmids from *Salmonella* isolates collected in Hubei province in 2015 and Zhejiang province in 2018 in China. We also noticed that an IncHI2 plasmid carrying *bla*_CTX-M-55_ and *qnrS1* was reported in Cambodia in 2016 [40]; this plasmid was identical or highly similar to the 278-kb one found in *Salmonella* Goldcoast. Therefore, it is very likely that the 278-kb conjugative plasmid was transferred to Taiwan from other Asian countries.

The 278-kb resistance plasmid harbored a high density of ISs and transposons that encompass not only *bla*_CTX-M-55_ and *qnrS1* but also a wide variety of other AMR genes. Further dissemination of the 278-kb conjugative plasmid into other *Salmonella* serotypes or even other Enterobacteriaceae species would make ciprofloxacin and ceftriaxone no longer effective for the treatment of infections and lead to a more frequent use of carbapenems, a drug of last resort. The use of antimicrobials in clinical practice and, more importantly, in food source livestock, would create a selective pressure that pushes AMR genes-carrying ISs and transposons to enter the plasmid, or for the entire conjugative plasmid to be transferred into susceptible bacteria.

## CONCLUSIONS

In this study, we have observed that Salmonella Goldcoast isolates in Europe and North America were much more susceptible than those in Taiwan and China because the isolates generally lack of ISs, transposons, or plasmids. The difference may be due to a more stringent control of antimicrobial use in patients as well as in food source livestock in Europe and North America.

## Supplementary Data

Supplementary materials are available at *Open Forum Infectious Diseases* online. Consisting of data provided by the authors to benefit the reader, the posted materials are not copyedited and are the sole responsibility of the authors, so questions or comments should be addressed to the corresponding author.

ofz447_suppl_Supplementary_Figure

ofz447_suppl_Supplementary_Table_S1

ofz447_suppl_Supplementary_Table_S2

ofz447_suppl_Supplementary_Table_S3

ofz447_suppl_Supplementary_Table_S4

ofz447_suppl_Supplementary_Table_S5
