# Supplementary figures and images for: Emergence and Evolution of High-Level Cephalosporin-Resistant Salmonella Goldcoast in Northern Taiwan
Source: Open Forum Infect Dis. 2019 Dec 17;6(12):ofz447. doi: 10.1093/ofid/ofz447 (PMC6916519; doi:10.1093/ofid/ofz447)

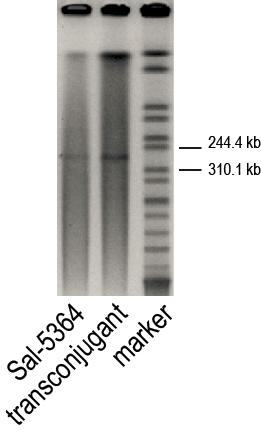

Supplement: ofz447_suppl_Supplementary_Figure [file ofz447_suppl_supplementary_figure.png]
