# Supplementary material for: Emergence and Evolution of High-Level Cephalosporin-Resistant Salmonella Goldcoast in Northern Taiwan
Source: Open Forum Infect Dis. 2019 Dec 17;6(12):ofz447. doi: 10.1093/ofid/ofz447 (PMC6916519; doi:10.1093/ofid/ofz447)
Supplement: ofz447_suppl_Supplementary_Table_S3 [file ofz447_suppl_supplementary_table_s3.docx]

**Supplementary Table 3. Age distribution of the patients and *S.* Goldcoast**

**isolation sources during the outbreak.**

| Age (year) | N (%) | Sample source | | |
| --- | --- | --- | --- | --- |
|  |  | Stool | Blood | Urine |
| < 1 | 7 (23.3%) | 7 (100%) | 0 (0) | 0 (0) |
| 2~5 | 7 (23.3%) | 6 (85.7%) | 1 (14.3%) | 0 (0) |
| 6~18 | 0 (0) | 0 (0) | 0 (0) | 0 (0) |
| 19~50 | 4 (13.3%) | 4 (100) | 1 (25%) | 0 (0) |
| 51~65 | 8 (26.7%) | 6 (75%) | 2 (25%) | 0 (0) |
| > 65 | 4 (13.3%) | 3 (75%) | 0 (0) | 1 (25%) |
