# Supplementary material for: Emergence and Evolution of High-Level Cephalosporin-Resistant Salmonella Goldcoast in Northern Taiwan
Source: Open Forum Infect Dis. 2019 Dec 17;6(12):ofz447. doi: 10.1093/ofid/ofz447 (PMC6916519; doi:10.1093/ofid/ofz447)
Supplement: ofz447_suppl_Supplementary_Table_S4 [file ofz447_suppl_supplementary_table_s4.docx]

**Supplementary Table 4. Clinical characteristics between adults and children.**

|  | Adults (N=16) | Children  (N=14) | *P* value |
| --- | --- | --- | --- |
| Admission | 15 (93%) | 12 (90%) | 0.81 |
| Hospital stay (days) | 15.4 ± 9.8 | 5.8 ± 4.7 | 0.002 |
| Fever | 7 (40%) | 8 (60%) | 0.79 |
| Duration of fever (days) | 1.4 ± 1.6 | 2.8 ± 2.2 | 0.07 |
| Diarrhea | 12 (76%) | 12 (86%) | 0.76 |
| Duration of diarrhea (days) | 2.1 ± 1.6 | 3.3 ± 3.4 | 0.35 |
| Frequency of diarrhea (times/ day) | 4.5 ± 4.9 | 8.0 ± 2.5 | 0.16 |
| Abdominal pain | 7 (40%) | 4 (30%) | 0.74 |
| Bloody stool | 0 (0%) | 5 (40%) | 0.62 |
