# Supplementary material for: Emergence and Evolution of High-Level Cephalosporin-Resistant Salmonella Goldcoast in Northern Taiwan
Source: Open Forum Infect Dis. 2019 Dec 17;6(12):ofz447. doi: 10.1093/ofid/ofz447 (PMC6916519; doi:10.1093/ofid/ofz447)
Supplement: ofz447_suppl_Supplementary_Table_S5 [file ofz447_suppl_supplementary_table_s5.docx]

**Supplementary Table 5. *Salmonella* isolated from 259 food samples obtained from traditional markets and supermarkets in northern Taiwan during Jan. to Oct., 2018.**

| **Food** | **Positive Rate** | **Serotype (n)** |
| --- | --- | --- |
| Pork | 55.3% (42/76)* | *S.* Agona (10)  *S.* Anatum (5)  *S.* Corvallis (1)  *S.* Derby (7)  *S.* Give (3)  *S.* Goldcoast (1)  *S.* Kentucky (1)  *S.* London (4)  *S.* Mbandaka (1)  *S.* Muenster (2)  *S.* Newport (2)  *S.* Potsdam (1)  *S.* Rissen (1)  *S.* Typhimurium (1)  *S.* Weltevreden (2) |
| Chicken | 42.1% (24/57) | *S.* Albany (3)  *S.* Anatum (3)  *S.* Brancaster (2)  *S.* Enteritidis (4)  *S.* Goldcoast (1)  *S.* Kentucky (4)  *S.* Livingstone (1)  *S.* Muenster (2)  *S.* Schwarzengrund (1)  *S.* Thompson (1)  *S.* Typhimurium (2) |
| Beef | 0% (0/20) | ND† |
| Pig intestine | 0% (0/2) | ND |
| Egg | 0% (0/28) | ND |
| Vegetables | 7.3% (3/41) | *S.* Derby (1)  *S.* Kaitaan (1)  *S.* Zigong (1) |
| Fruits | 0% (0/14) | ND |
| Seafood | 0% (0/13) | ND |
| Delicatessen | 0% (0/8) | ND |

*The number of *Salmonella* isolated/the number of the sample examined. †ND, not detected.
